# LAPTM4B-35 protein is a weak tumor-associated antigen candidate

**DOI:** 10.3892/etm.2013.1427

**Published:** 2013-11-26

**Authors:** GUILAN SHI, CHUNXIA ZHOU, DONGMEI WANG, WENBO MA, SHUREN ZHANG

**Affiliations:** 1 Department of Immunology, Zibo Vocational Institute, Zibo, Shandong 255314, P.R. China; 2 Department of Immunology, Cancer Institute, Peking Union Medical College and Chinese Academy of Medical Sciences, Beijing 100021, P.R. China

**Keywords:** LAPTM4B-35, LAPTM4B-24, tumor-associated antigen, immunogenicity

## Abstract

Lysosome-associated protein transmembrane 4β (LAPTM4B) is a gene that has been indicated to be involved in cancer. It is located at chromosome 8q22 and is composed of seven exons and six introns. LAPTM4B encodes two protein isoforms: LAPTM4B-35 and LAPTM4B-24. LAPTM4B-35 is markedly upregulated and LAPTM4B-24 is downregulated in several types of cancer. LAPTM4B-35 is 91 amino acids (N91) longer than LAPTM4B-24 at the N-terminus. In the present study, western blotting, enzyme-linked immunosorbent spot analysis and the B16F10-N91 tumor bearing-mice experiments were used to evaluate whether the overexpression of N91 indicates its potential as a candidate tumor-associated antigen. The results revealed that N91 was expressed in a wide range of normal mouse tissues and human peripheral blood mononuclear cells, with varying expression levels. The weak immunogenicity of N91 protein suggested it was a weak candidate antigen; however, the N91 protein was associated with cell proliferation.

## Introduction

The characterization of tumor-associated antigens (TAAs), recognized by cellular or humoral effectors of the immune system, has provided novel perspectives for cancer therapy (1). Immunomodulation strategies, such as peptide-based approaches or gene vaccines, are considered to be potential adjuvant therapies in patients with cancer, either to treat minimal residual disease or to prevent relapse. These strategies are based on the hypothesis that the T-cell repertoire of an individual contains TAA-primed memory T cells, and that the patient’s immune system is capable of being sensitized to the TAAs of the patient’s own tumor (2).

The lysosome-associated protein transmembrane 4β (LAPTM4B) gene contains two translation initiation codons, separated by 273 bp, which encode two protein isoforms: LAPTM4B-35 and LAPTM4B-24, with molecular weights of 35 and 24 kDa, respectively. LAPTM4B-35 is 91 amino acids (N91) longer than LAPTM4B-24 at the N-terminus. Previous studies have shown that LAPTM4B-35 is overexpressed in a number of malignant tissues and has a significant correlation with the prognosis of several types of cancer, such as hepatocellular (3) and cervical carcinoma (4), breast cancer (5), endometrial carcinoma (6) and ovarian cancer (7). In addition, the LAPTM4B gene has been demonstrated to promote cell proliferation by regulating cell cycle control and causing tumorigenesis of NIH3T3 cells, indicating that it is important in tumorigenesis (8). Furthermore, LAPTM4B-35 promotes the multidrug resistance of cancer cells (9). By contrast, LAPTM4B-24 is downregulated in several types of cancer (8,10–12). Thus, N91 may be a potential candidate for an overexpressed TAA. However, little is known about the incidence and magnitude of a pre-existing tumor-specific cellular immune response against N91 protein in patients with cancer. Therefore, the aim of the present study was to evaluate the potential of N91 protein as a TAA to induce an antitumor immune response.

## Materials and methods

### Cell lines, animals and blood samples

The human tumor cell lines HepG2 (HLA-A*0201^+^), HeLa, MCF7, Skov3 and T2 (HLA-A*0201^+^) were maintained in the Department of Immunology, Cancer Institute, Peking Union Medical College and Chinese Academy of Medical Sciences (Beijing, China). These human tumor cells were maintained in RPMI-1640 medium containing 10% heated-inactivated fetal calf serum, 2 mM L-glutamine, 10 mM HEPES, penicillin (100 U/ml)-streptomycin (50 μg/ml) solution and 1% sodium pyruvate solution.

Female C57BL/6 and Balb/c mice were purchased from the Experimental Animal Institute of Peking Union Medical College (Beijing, China) and maintained in a specific pathogen-free environment. The mice were ready for experimental use at six to eight weeks of age. The Animal Research Ethics Committee of the Cancer Institute and Hospital, Peking Union Medical College and the Chinese Academy of Medical Sciences (no. 20120005; Beijing, China) approved all the protocols involving animals.

Peripheral blood mononuclear cell (PBMC) samples were obtained from 67 patients with hepatic carcinoma, cervical carcinoma, breast cancer or ovarian cancer, prior to surgery at the Cancer Institute and Hospital (Beijing, China) between January 2009 and February 2012. The patient population comprised 41 males and 26 females, with a mean age of 52.64 years (range, 23–81 years). In addition, 25 blood samples were obtained from healthy donors (19 males and 6 females; median age, 31 years). The Institutional Ethics Committee of Peking Union Medical College approved the study prior to its initiation, and written informed consent was provided by all the participants.

### Peptide synthesis and HLA-A*0201 peptide-stabilization assay

The HLA-A*0201-binding peptides in the N91 sequence were identified using the publicly available peptide-motif scoring systems (http://www-bimas.cit.nih.gov/molbio/hla_bind/ and http://www.syfpeithi.de). The potential natural processing of the peptides by proteasomal cleavage was evaluated using the Prediction Algorithm for Proteasomal Cleavages website (http://www.paproc.de). The following three peptides were identified: GLQARRSTL (N91-1), PLPVPAAAAV (N91-2) and QARRSTLLKTC (N91-3). The peptides were synthesized by Sangon Biotech Co., Ltd. (Shanghai, China) and purified (>95%) using high-performance liquid chromatography. The synthesized peptides were maintained in dimethylsulfoxide (DMSO), aliquoted at 10 mg/ml and stored at −80ºC.

Each peptide was examined for the ability to undergo concentration-dependent binding to a transporter associated with antigen processing-defective cells (T2) in an HLA-A*0201 stabilization assay (13,14). The T2 cells were incubated at room temperature overnight, with peptide concentrations of 50–100 μg/ml, in AIM-V medium (GIBCO^®^ Life technology™, Grand Island, NY, USA) containing 5 μg/ml β2-microglobulin. Following staining with HLA-A2-specific monoclonal antibody (mAb; clone BB7.2, conjugation of PE; Biolegend, San Diego, CA, USA), flow cytometry was used to assess the stability of HLA-A*0201. A phycoerythrin (PE)-labeled isotype-matched antibody (mouse IgG2b; Biolegend) was used as the control. The HLA-A*0201 standard binding peptide, Flu (GILGFVFTL), was used as a positive control and Msurv33 (LYLKNYRIA), from the H-2d mouse, was used as a negative control (15). Data were expressed as an increase in mean fluorescence intensity (MFI) of the cells, with each peptide compared with the cells without peptide or with a negative control peptide [FI = (MFI of T2 cells + peptide)/(MFI of T2 cells with negative peptide)]. FlowJo software (Tree Star, Inc., Ashland, OR, USA) was used to analyze the acquired data.

### Generation of antigen-specific cytotoxic T lymphocytes (CTLs)

The glutathione S-transferase (GST)-N91 fusion protein (supplied by RL Zhou from the Department of Cell Biology, Peking University Health Science Center, Beijing, China) was purified by affinity chromatography using GST™ resin (Merck KGaA, Darmstadt, Germany), in accordance with the manufacturer’s instructions. SDS-PAGE was performed to analyze the purified protein from the infected cells. In brief, the separating gel was 12.5% w/v and the stacking gel was 4% w/v. Prior to electrophoresis, the samples were heated in the presence of sample buffer at 100ºC for 5 min in a boiling water bath. Each lane contained 40 μg of protein extraction. Electrophoresis was performed using a constant voltage of 80 V for 95 min. Proteins were visualized in gels by staining with 0.025% Coomassie brilliant blue (R-250, CBB; Sigma, St. Louis, MO, USA) in 50% (v/v) methanol, 10% (v/v) acetic acid. The GST-N91 fusion protein was then digested with PreScission Protease (GE Healthcare Life Sciences, Piscataway, NJ, USA) in cleavage buffer. Following the completion of the digestion, Glutathione Sepharose was added to the sample to remove the GST protein. The N91 protein was stored at −80ºC for use.

PBMCs were isolated using Ficoll-Paque™ PLUS medium (GE Healthcare Life Sciences). In brief, the diluted blood sample was carefully layered on Ficoll-Paque PLUS, then centrifuged at 400 × g for 20–30 min at 18–20ºC. The undisturbed lymphocyte layer was obtained at the interface. After washing with phosphate buffer saline, the lymphocytes were used as PBMC sources. The PBMCs and tumor cell lines were screened for HLA-A*0201 expression by flow cytometry using an HLA-A2-specific monoclonal antibody (mAb; clone BB7.2; BioLegend). The genotyping for the HLA-A2 alleles was performed using reverse transcription-polymerase chain reaction (RT-PCR) amplification with sequence-specific primers and sequence-based typing (16). The HLA-A*0201 subtyping was detected using ThermoScript ™ Reverse Transcriptase kits (Invitrogen™ Life Technologies, Grand Island, NY, USA) according to the manufacturer’s instructions. The sequence of primers and the primer combination were shown in Tables I and II, respectively. The healthy donors and patients were selected on the basis of HLA-A*0201 antigen expression (data not shown). Monocyte-derived dendritic cells (DCs) were generated from PBMCs (HLA-A*0201) as previously described (17). Mature DCs (mDCs) exhibited the phenotype CD14^low^, CD11c^+^, CD86^high^, HLA-DR^high^ and MHC-I^high^. mDCs were pulsed with 50 or 100 μg/ml control peptide or 20 μg/ml N91 protein in serum-free AIM-V medium overnight.

To induce CTLs *in vitro*, the PBMCs (2×10^6^/ml) were suspended in serum-free medium and divided into two fractions. One of the fractions was cultured with a pool of the three N91 peptide-pulsed DCs, while the other fraction was incubated with N91 protein-pulsed DCs (2×10^5^/ml) in 6-well plate (1 ml per well). The CTLs were generated by two cycles of stimulation with peptide or N91 protein-loaded DCs every seven days (DC:PBMC = 1:10) (18). The CTL functions were analyzed using interferon-γ (IFN-γ) enzyme-linked immunosorbent spot (ELISPOT) assay kits (BD-Pharmingen, San Jose, CA, USA), in accordance with the manufacturer’s instructions. Typically, a fixed number of various target and effector cells (5×10^4^ cells per well, effector to HepG2 target ratio of 40:1) were cultured in replicate wells overnight, and the spots were quantified using an immunospot reader (Cellular Technology Limited, Shaker Heights, OH, USA). The results were presented as the number of IFN-γ-producing cells per 5×10^4^ cells.

### Tissue collection, protein extracts and western blot analysis

The tissue samples from the C57BL/6 mice were flash-frozen in liquid nitrogen immediately subsequent to collection and lysed in radioimmunoprecipitation assay (RIPA) lysis buffer. PBMCs or splenocytes from the C57BL/6 and Balb/c mice were activated with phytohemagglutinin (5 μg/ml) or concanavalin-A (4 μg/ml) for 72 h, respectively. The cells were then harvested and lysed in prechilled RIPA lysis buffer. Following centrifugation at 10,000 × g for 15 min at 4ºC, the supernatant was collected for western blotting. Protein concentration was analyzed by monitoring the visible spectrophotometer absorbance at 595 nm. For western blotting, the protein was separated using 12.5% SDS-PAGE, and the proteins were transferred onto Immobilon-FL polyvinylidene difluoride (PVDF) membranes (Millipore, Billerica, MA, USA) using a wet-transfer apparatus (Bio-Rad, Hercules, CA, USA). The membranes were subsequently blocked by incubation in 5% non-fat dry milk, washed and incubated with specific antibody [rabbit immunoglobulin G (IgG), supplied by RL Zhou], followed by secondary reagents [anti-rabbit IgG labeled with horseradish peroxidase (HRP); Cell Signaling Technology, Danvers, MA, USA]. The membranes were visualized using an enhanced chemiluminescence (ECL) western blotting system (ECL western blotting system, GE Healthcare Life Sciences). As an internal loading control, β-actin was probed with a commercial antibody (Cell Signaling Technology, Inc.).

### Statistical analysis

Data were expressed as the mean ± standard error of the mean (S.E.). Differences between groups were evaluated using one-way analysis of variance (ANOVA). P<0.05 was considered to indicate a statistically significant difference. Statistical analyses were performed using the commercially available software SPSS 16.0 (SPSS, Inc., Chicago, IL, USA).

## Results

### Stabilization of HLA-A*0201 on a T2 cell line

**Figure 1:**
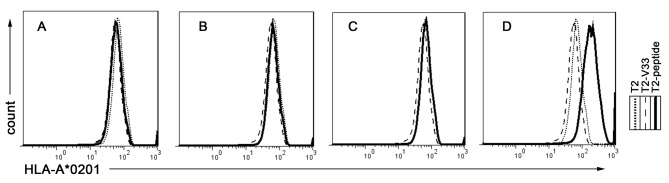
Stabilization of surface HLA class I molecules by synthetic peptides. T2 cells were either left untreated or pulsed with the indicated synthetic peptides at 50–100 μg/ml and human β2-microglobulin at 5 μg/ml. Following overnight incubation, the cells were treated with either phycoerythrin (PE)-labeled isotype-matched control antibody or BB7.2 antibody, and subjected to flow cytometry. The graph shows the mean fluorescence intensity (MFI) of peaks representing untreated or peptide-pulsed T2 cells. (A–C) MFI of HLA-A2 on T2 cells pulsed with different N91 peptides (A, N91-1; B, N91-2; C, N91-3) compared with untreated T2 cells or cells treated with negative control peptide. (D) T2 cells pulsed with positive control peptide (Flu), which stabilized the HLA-A2 molecules, as shown by the shift in the peak. V33, Msurv33.

To assess whether synthetic peptides were able to stabilize the expression of the HLA-A*0201 molecule on the T2 cell surface, peptide-induced HLA-A*0201 upregulation on the T2 cells was examined according to a previously described protocol (19). When three different peptides of the N91 sequence (Table III) were co-cultured with the T2 cells, the peptides weakly enhanced the surface expression of HLA-A*0201 (Fig. 1A–C). However, pulsing the cells with the positive control peptide (Flu) notably stabilized the HLA-A*0201 expression on the surface of the T2 cells (Fig. 1D). The binding data are shown in Table I: All three examined peptides were observed to weakly stabilize HLA-A*0201. In general, the MHC stabilization by these peptides was more consistent with the calculated score based on the half-life of dissociation of the peptide from the HLA molecule than the binding affinity as calculated by the SYFPEITHI epitopes prediction.

### CTL responses against peptides or N91 protein-loaded targets

**Figure 2:**
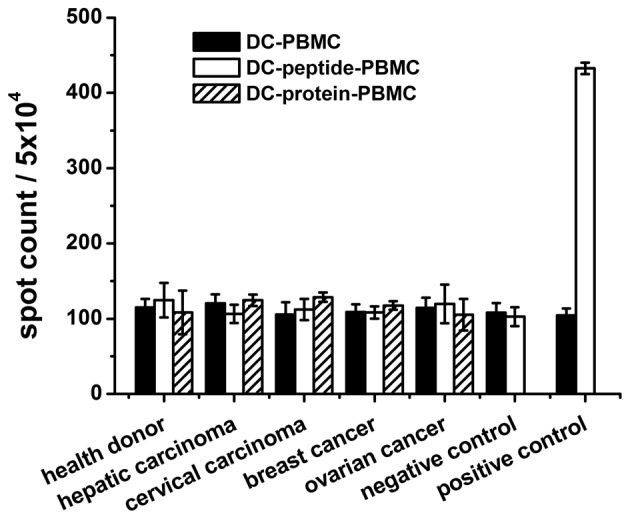
Enzyme-linked immunosorbent spot (ELISPOT) assay. Cytotoxic T-cell responses, assayed using an interferon-γ (IFN-γ) spot assay, against N91 peptides or HepG2 tumor cells. A fixed number of various target and effector cells (5×10^4^ cells per well, effector to HepG2 target ratio of 40:1) were cultured in replicate wells overnight. PBMC, peripheral blood mononuclear cell.

It was assessed whether the peptides (N91-1 one of the 3 peptides tested, amino acid sequence is ‘GLQARRSTL’) or N91 protein were able to bind to HLA-A*0201^+^ DCs and induce a CTL response against the target cells (HepG2 and HLA-A*0201^+^) loaded with N91 protein. The responses of PBMCs to N91 peptide or protein were examined in 67 HLA-A*0201^+^ patients, while the frequency of the T-cell response to N91 peptide or protein, positive Flu peptide and the negative control, V33 peptide, was evaluated in healthy donors. The ELISPOT assay was considered to be the most sensitive and feasible screening test for the N91-specific T-cell assay. As shown in Fig. 2, the activation of CTLs was undetectable or negligible. No reactivity was observed for the N91 peptide or protein in the patients or healthy donors. The B16F10-N91 tumor-bearing mice experiments demonstrated that the N91 specific immune response was also negligible (data not shown).

### Expression level of the N91 protein in normal and cancer cells

**Figure 3:**
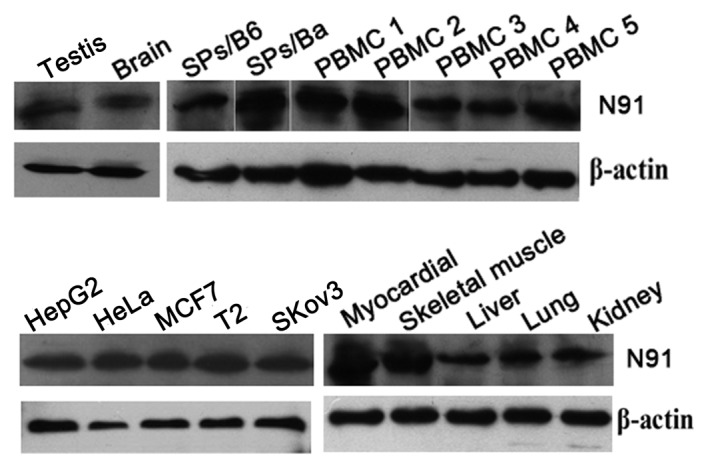
Analysis of the expression levels of N91 in human tumor cell lines and normal tissue. Tissue samples from C57BL/6 mice were flash-frozen in liquid nitrogen immediately subsequent to collection and lysed in lysis buffer. Activated peripheral blood mononuclear cells (PBMCs) and splenocytes from C57BL/6 (SPs/B6) or Balb/c (SPs/Ba) mice were harvested and lysed. For western blotting, 45 μg protein was separated using 12.5% SDS-PAGE).

The previously described experiments showed that CTLs against N91 protein were not able to be induced *in vitro*. To elucidate the mechanism underlying this phenomenon, the expression levels of N91 proteins from normal tissues or cells were examined using western blot analysis. Human LAPTM4B shares 92% homology with mouse LAPTM4B at the amino acid level, suggesting that LAPTM4B has been highly conserved within vertebrate species (8). Thus, the expression level of the N91 protein was assessed in mouse tissues. As shown in Fig. 3, N91 protein expression was detected by western blotting in certain tumor cell lines and normal tissues, including the lysates of activated PBMCs from normal healthy donors, lysates of the liver, kidney, brain, testis, skeletal muscle and myocardium, and activated splenocytes from naïve C57BL/6 and Balb/c mice. No significant differences were identified between the expression levels of N91 protein in the tumor cell lines and the normal tissue. Thus, it was suggested that the widespread expression of the N91 protein may be responsible for the weak MHC class I processing and epitope presentation.

## Discussion

The majority of cancer antigens are ‘self-antigens’, expressed on normal cells. Immunogenic peptides derived from these TAAs have been used in therapeutic vaccines (20,21). Synthetic peptides from the predicted high-binding category are loaded on HLA-matched DCs or other antigen-presenting cells, and used as stimulators in CTL induction. The LAPTM4B-35 protein is significantly upregulated in several types of cancer (8), indicating that it may be a candidate tumor antigenic epitope. According to the SYFPEITHI analysis of the N91 protein sequence, three different peptides were synthesized. However, the results demonstrated that it was not possible to induce an immune response using peptide-pulsed DCs co-cultured with PBMCs *in vitro*, as examined using the IFN-γ assay. Previous studies with other cancer-specific peptides have demonstrated that CTL responses are able to be induced by peptides with various MHC binding affinities (22,23). Furthermore, it has been shown that tolerance to a self-peptide is most efficiently established by those self-peptides with a high-binding affinity for MHC (24). Alternatively, a lower binding affinity may induce an antitumor immune response to some degree. Thus, a N91 protein-pulsed DCs experiment was performed to investigate this. However, there no significant differences were identified between the DC-protein-PBMC and DC-PBMC groups. The high expression of the N91 protein in certain normal tissues and activated PBMCs, as well as in malignant cells, may have been responsible for the negative result. The results suggested that the overexpression of the N91 protein may not be a candidate for a TAA.

Certain aspects of currently available technologies inhibit the identification of novel tumor antigens. Differential genomics and proteomics approaches identify over- and underexpressed proteins. However, these methods are unable to identify very low-abundance proteins that are often processed and presented by MHC class I molecules as the true rejection targets for T cells. Furthermore, the level of protein expression does not always correlate with MHC processing and presentation in cancer (25). In addition, a study of tumor immunology suggested that tumor development is associated with the induction of tolerance to tumor antigens, leading to immunosuppression, which prevents an autologous adoptive immunotherapy for patients with refractory metastatic solid tumors (26). In patients with cancer, spontaneous antitumor immune responses appear to be too weak to cause the spontaneous rejection of the tumors, since the cancer continues to develop (27). A major concern for the use of tumor-derived antigens is the possibility that the epitope may activate autoimmune disease. Furthermore, many of these peptides are not tumor-restricted. This may underlie the mechanism whereby the overexpression of the N91 protein is not recognized by DCs as a tumor antigen.

Therefore, the identification of novel TAAs remains one of the major aims for the design of more effective immunological treatments for cancer. Ideal targets for immunotherapy are gene products that are silenced in normal tissues and only overexpressed in cancer cells, and that are directly involved in tumor survival and progression. The weak immunogenicity of N91 protein may be explained by its increased expression in certain normal tissues, as well as malignant cells. Thus, it appears to be a weak candidate for a TAA.

## Figures and Tables

**Table I:** Primers for the detection of the HLA-A*0201.

Primer	Sequence (5′-3′)
Genomic primers	
Sense-primer	
AL#37	CCT CGT CCC CAG GCT CT
Antisense-primer	
AL#AW	TGG CCC CTG GTA CCC GT
Nested primers	
Sense-primer	
AL#22	CAC TCC ATG AGG TAT TTC TT
AL#14	AGG CCC ACT CAC AGA CTC
AL#3	GAC GGG GAG ACA CGG AAA
Antisense-primer	
AL#Q	CTC CAG GTA GGC TCT CAA
AL#BG	CGT CGC AGC CAT ACA TCC
AL#BF	CCC CAC GTC GCA GCC AT
AL#V	GAG CCA CTC CAC GCA CGT

**Table II:** Reaction combinations for the detection of the HLA-A*0201.

Primer combination	Product size (bp)
Step1	
AL#37, AL#AW	812
Step2	
AL#22, AL#Q	718
AL#22, AL#BG	542
AL#22, AL#BF	547
AL#14, AL#V	543
AL#3, AL#V	565

**Table III:** HLA-A*0201 epitope affinity assay.

Peptide	Amino acid sequence	Fluorescence intensity
N91-1	GLQARRSTL	0.98
N91-2	PLPVPAAAAV	0.90
N91-3	QARRSTLLKTC	0.90
Influenza (Flu)	GILGFVFVFTL	2.70
Msurv33 (V33)	LYLKNYRIA	0.90

Flu and V33 peptides were positive and negative controls, respectively. Fluorescence intensity (FI) = mean FI (MFI) of T2 cells with peptide/MFI of T2 cells with negative peptide.
